# Environmental Pollution in Geopark Management: A Systematic Review of the Literary Evidence

**DOI:** 10.3390/ijerph19084748

**Published:** 2022-04-14

**Authors:** Anna V. Mikhailenko, Natalia N. Yashalova, Dmitry A. Ruban

**Affiliations:** 1 Department of Physical Geography, Ecology, and Nature Protection, Institute of Earth Sciences, Southern Federal University, Zorge Street 40, 344090 Rostov-on-Don, Russia; avmihaylenko@sfedu.ru; 2 Department of Economics and Management, Business School, Cherepovets State University, Sovetskiy Avenue 10, 162600 Cherepovets, Vologda Region, Russia; natalij2005@mail.ru; 3 K. G. Razumovsky Moscow State University of Technologies and Management (The First Cossack University), Zemlyanoy Val Street 73, 109004 Moscow, Russia; 4 Department of Organization and Technologies of Service Activities, Higher School of Business, Southern Federal University, 23-ja Linija Street 43, 344019 Rostov-on-Don, Russia

**Keywords:** environmental management, geotourism, landscape pollution

## Abstract

Dozens of geoparks have been created in the world since the beginning of the 21st century. Their environmental impact is yet to be fully understood. A bibliographical survey was undertaken to systematically review the journal articles devoted to environmental pollution in geoparks. The considered literature focuses on 10 geoparks (many of them are the members of the UNESCO Global Geoparks network) from eight countries, namely, China, Italy, Malaysia, Poland, Portugal, Romania, Russia, and South Korea. Significant pollution was registered in half of these geoparks. Trace metals and metalloids such as arsenic and cadmium are often reported as pollutants. Water pollution is the most common. In many cases, environmental pollution is not related to geoparks, but results from agricultural and industrial activities. Sometimes, this pollution is inherited from past mining activities, and the latter are related to the geoheritage represented in the geoparks. However, there are also examples of pollution triggered by tourist activities in geoparks and the related infrastructural failures. Various mitigation approaches are considered in the literature (establishing monitoring networks, installing filtration membranes, etc.). It is argued that environmental pollution can be used in geoparks for eco-education and eco-awareness initiatives. Research in environmental pollution in geoparks is an emerging field, and does not avoid multiple biases. Nonetheless, the actual importance of this research is undisputable, and it will be demanded in the future.

## 1. Introduction

Environmental pollution is typical of not only industrial and densely populated territories [1,2,3], but also tourism-important landscapes [4,5,6] and protected areas [7,8]. A novel category of public establishments, which share touristic and conservation features, has appeared recently, namely, geoparks [9,10,11,12,13]. Generally, geoparks are based on unique geological sites (geoheritage sites) and hosting landscapes, and they serve the purposes of geotourism [14], geo-education [15], and local sustainable development [16]. The noted sites are diverse—for instance, they include famous dinosaur localities, geysers, historical mining sites, stratotype sections, and unusual landforms. Many geoparks are included in national and international geopark networks. The most important geoparks, which represent the most valuable portions of the world’s geoheritage and put geotourism development on the international level, constitute the UNESCO Global Geoparks network—a valuable initiative contributing to global sustainable development [10]. At the beginning of 2022, this network included 169 geoparks from 44 countries, with a limited number of transnational (transboundary) geoparks [17].

**Figure 1:**
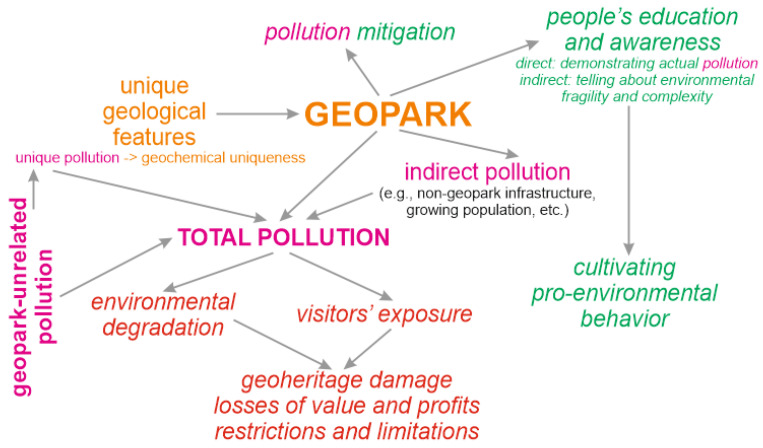
Conceptual representation of pollution in geoparks (see text for explanations); negative consequences are highlighted in red and positive consequences are highlighted in green.

There are three main reasons to pay close attention to environmental pollution in geoparks. First, geoparks themselves are human interventions in natural landscapes with inevitable environmental stress. It should be underlined that geopark creation and the world’s geotourism growth are not only closely related, but are also mutually facilitating phenomena [18]. As tourism triggers significant environmental pollution [4,5,6,18,19,20,21,22,23], geoparks can be regarded as territories with high risks for nature. Geoparks represent the geological environment, pollution of which challenges its balanced use [24]. Additionally, some geoparks utilize abandoned mining sites, which are known for their pollution [25]. Even if such pollution is unrelated to geoparks themselves, it can be inherited by geoparks. Second, geoparks are tourist attractions, and, thus, pollution in them increases people’s exposure to dangerous substances. This situation is typical for tourist destinations (for instance, [26]). Third, chemical pollution of a given geological landscape can be unique because of its scale or specific processes; in such cases, the related features are valuable and important for eco-education initiatives [27,28,29]. Indeed, the potential of geoheritage sites for learning sustainability [30,31] should be used. Generally, pollution in geoparks may be related to their functioning, it can constitute a highly specific geopark resource, and it has both negative and, surprisingly, positive consequences (the latter matter only when this pollution is manageable) (Figure 1). Indeed, environmental pollution in geoparks should be distinguished from the other forms of anthropogenic pressure, including physical destruction of unique objects such as natural outcrops or speleothems, uncontrolled litter accumulation, and artificial landscape re-organization. Furthermore, a comprehensive conceptualization of environmental pollution in geoparks is lacking, and a pioneering summary of the lines of evidence is urgently needed.

Although geoparks are rather new establishments, sustainability-related issues are crucial to them, and, thus, it is unsurprising that some research aimed at environmental pollution on their territories has been undertaken. The present and future effective management of geoparks requires deeper understanding of the issue (see the three reasons above). However, the currently available knowledge is scattered throughout a large amount of literature, including environmental, geographical, and geological journals; and this knowledge is also fragmented and needing serious conceptualization.

The objective of the present work is to gather small pieces of the scientific information about environmental pollution in geoparks in order to check its state critically and systematically, to detect biases, and to offer a tentative vision for this research direction. The latter seems to be highly important in regard to the ongoing expansion of the geopark networks and acceleration of geotourism. Essentially, this work is a systematic review of the literary evidence. Indeed, it is not and cannot be a comprehensive review of the entire issue of environmental pollution in geoparks because the related knowledge is too fragmented and often inaccessible. However, this literature review, aimed at generalizing the views of scholars, is an initial step towards such a comprehensive understanding of the issue. At least, such systematic reviews of the scattered literary evidence allow the establishment of tentative frameworks for further accumulation of information from different sources. Taking into account the novelty of the considered issue, the focus on the published research outcomes seems to be a reasonable approach to lay the foundation for further developments.

## 2. Bibliographical Survey

Methodologically, the present work is a bibliographical survey aimed at reviewing the issue of environmental pollution in geoparks on the basis of the published information. Such systematic reviews, coupled sometimes with bibliometric analyses, have been proven to be efficient research instruments for effective summarizing, critical assessment, and conceptualization of the previously accumulated but scattered information [5,32,33,34,35]. In this study, only journal articles published in international journals were considered to ensure the set of selected sources was representative (conference volumes, books, and publications in languages other than English were excluded because, first, it is technically impossible to collect the majority of these, and, second, articles in well-established, peer-reviewed journals are trusted and reflect the international state of research). Certain incompleteness is unavoidable in studies such as this, but this can be taken into account in further interpretations (see the Conclusion of this paper).

**Figure 2:**
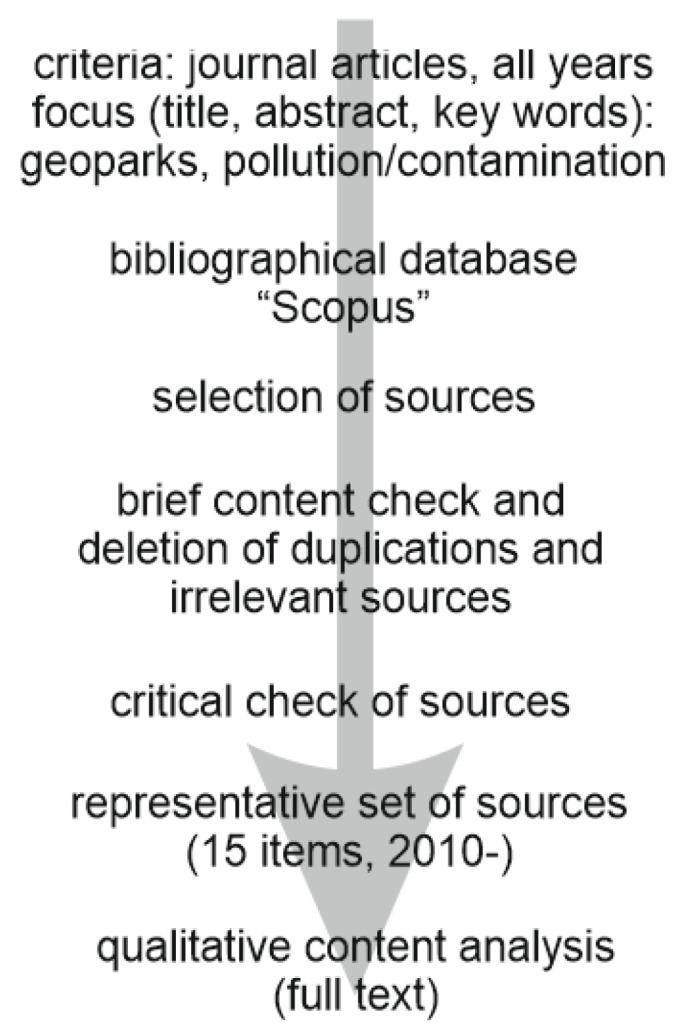
Procedures of the present bibliographical survey.

Journal articles were selected for the purposes of this review in several steps (Figure 2). In order to identify the proper sources, the bibliographical database “Scopus” was used due to its exceptional coverage of the scientific publications [36]. Importantly, acceleration of the geopark-related research began very recently [11,37], and, thus, it matches the period for which the database coverage is the best. This database allows finding sources containing such words as “environment(al)”, “pollution”, “contamination”, and “geopark(s)” in their titles, abstracts, and key words. Then, the “automatically” collected sources were checked “manually” in order to avoid duplications or irrelevant sources. The remaining sources pay attention to environmental pollution in geoparks and represent the available literature dealing with this important issue. The relatively limited number of such journal articles was expected because this research direction is highly specific and very new. The oldest source was published in 2010, and, thus, the decade-long duration of research in environmental pollution in geoparks generally matches the rising scientific interest in geoparks.

The content of the collected literature (full text of each source) was analyzed qualitatively and systematically. Attention was paid to four principal aspects of the present knowledge of environmental pollution in geoparks. First, the geographical focus of the collected articles was reviewed, with a specific focus on the number of members of the UNESCO Global Geoparks network for which pollution has been a concern. Second, the information about the established pollutants and their occurrence in landscape components (air, soil, water, biota, etc.) was summarized. Although one may suppose that pollution of the geological environment would the most common target of research, it should be recalled that geoparks represent landscapes, i.e., they include not only geological and geomorphological features, but also water objects, vegetation, etc. Third, the possible sources of pollution were addressed. In regard to the conceptual scheme proposed above (Figure 1), it appears crucial to understand whether the reported pollution is linked to geopark functioning or results from the other human activities and/or natural processes. Fourth, it is evident that studying environmental pollution requires its careful documentation, and the offering of recommendations for its mitigation and landscape rehabilitation. It is expected that some articles deal with this important aspect, and the related knowledge was summarized.

The methodological outline of this and many other similar works raises questions regarding simplicity and novelty. Systematic reviews of literary evidence have been proven to be full-scale research tools in many branches of contemporary science [36,38,39,40]. These permit accumulation of the already published information, in addition to its critical treatment, systematization, and conceptualization. The published sources (journal articles in this case) are the objects of analysis, which new conclusions to be reached about the state of research fields, research biases, and opportunities. Such reviews allow the foundation to be laid for further scientific developments. Finally, they serve as advanced references for newcomers to emerging research fields. In other words, their methodological complexity is determined by the systematization and reconsideration of the published information (i.e., the content of journal articles), and their true novelty is linked to the portions of conceptual information generated via qualitative (and sometimes quantitative) analyses of the available literature.

## 3. Review of Literary Evidence

### 3.1. Geographical Extent

**Figure 3:**
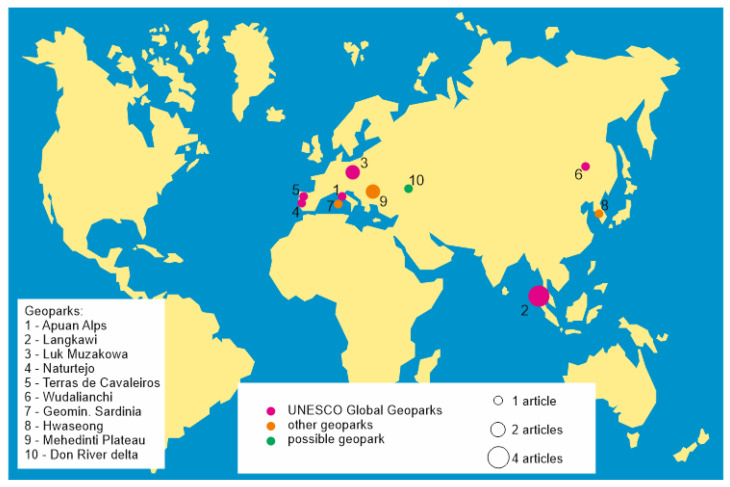
Geographical distribution of the considered geoparks.

The literature on environmental pollution in geoparks has a rather wide geographical focus (Figure 3). It deals with eight countries, namely China [41], Italy [42,43], Malaysia [44,45,46,47], Poland [48,49], Portugal [50,51], Romania [52,53], Russia [29], and South Korea [27]. Of these, the biggest attention has been paid to Malaysia, where environmental pollution in geoparks has been studied for about a decade by several research teams. It is notable that the analyzed literature focuses either on European or Asian geoparks, although geoparks have also been created in some other parts of the world.

A total of ten geoparks are considered in the analyzed literature. A share of 93% of the articles deal with the already established geoparks, and only Mikhailenko and Ruban [29] address a geopark proposal in Russia. A share of 60% of the considered geoparks are UNESCO Global Geoparks, including the Apuan Alps [42], Langkawi [44,45,46,47], Muskauer Faltenbogen/Łuk Mużakowa [48,49], Naturtejo [50], Terras de Cavaleiros [51], and Wudalianchi [41]. These global geoparks represent five European and Asian countries, of which Portugal hosts two geoparks studied in regard to environmental pollution. One geopark is transnational, belonging to both Germany and Poland, but the experts [48,49] paid attention to its only Polish part. The remaining 40% of the geoparks are not listed among the UNESCO Global Geoparks, although some of them are members of the other international (e.g., the European Geoparks) or national networks. These include such geoparks as the Geomining Park of Sardinia [43], Hwaseong [27], Mehedinti Plateau [52,53], and the possible geopark in the Don River delta [29]. These geoparks represent four countries, both European and Asian.

The available literary evidence implies that environmental pollution has been studied for less than 4% of all UNESCO Global Geoparks. However, one should note that several (if not many) of the latter are new and/or received their high status very recently. The time available to recognize the issue of environmental pollution, study it, and publish the results was not sufficient for many UNESCO Global Geoparks. If it is taken into account that this network included only 117 geoparks before 2017 [54], i.e., prior to the past five years, the share of those subject to environmental pollution studies increases to 5%. Although this number seems to be small at first glance, it is not too small to rule out the importance of environmental pollution studies in the UNESCO Global Geoparks. At least, it is evident that environmental pollution has emerged as an actual or potential problem for some of them. This is especially the case of the Langkawi geopark in Malaysia, which has been studied intensively regarding this aspect (see more details below).

### 3.2. Pollutants in Geopark Landscapes

Different patterns of environmental pollution (either existing or potential) have been established in the UNESCO Global Geoparks. In the Apuan Alps geopark (Italy), Ghezzi et al. [42] paid attention to a high-altitude vineyard. Some chemical peculiarities linked to its geological setting were revealed, and potentially toxic elements (arsenic, cadmium, copper, lead, and zinc) were found in small, non-dangerous concentrations. The issue of pollution does not exist in this case. The situation differs in the Langkawi geopark (Malaysia) where anthropogenic pressure seems to be really significant. Shamshiry et al. [47] documented stream and groundwater pollution by cadmium (0.08 mg/L), which exceeded the threshold concentrations by more than 10 times. Aris et al. [44] noted pollution of river water by organic compounds and various heavy metals. Ibrahim et al. [45] established that water is polluted with oils and litter, and these authors also documented noise pollution of the local landscapes. Importantly, this pollution was established on the basis of the opinions of locals, visitors, and geopark staff, i.e., this is a kind of perceived pollution. Finally, Mokhtar et al. [46] focused on marine coastal sediments, where pollution by arsenic (11.42 mg/kg dry weight) was found.

Two studies devoted to the Muskauer Faltenbogen/Łuk Mużakowa geopark [48,49] registered the absence of a negative influence of acid mine drainage on diatom and cladoceran assemblages in a small young lake created in a clay pit (apparently, clay bedrock prohibits pollution with heavy metals); however, they also found pollution of river bed sediments with aluminum (up to 993 ppm) and iron (up to 2655 ppm), which can be remobilized and become water pollutants. Albuquerque et al. [50] developed and tested a new approach for a groundwater vulnerability assessment. In this case, potential pollution of any kind was considered, and high vulnerability was established in some areas of the Naturtejo geopark. The Terras de Cavaleiros geopark hosts a closed mining complex, and the area was the subject of some rehabilitation. The study by Antunes et al. [51] established that stream sediments, soils, and artificial lake water are polluted by arsenic (up to 62.2 μg/L in surface waters) and wolfram (up to 1100 mg/kg in stream sediments), in addition to barium, tin, and other elements. The degree of pollution is so high that water should not be used for human consumption or even in agriculture. In the Wudalianchi geopark, Zhang et al. [41] identified pollution of mineral springs and groundwater by nitrates, with concentrations of NO_3_^−^ exceeding 0.5 mmol/L and even 1.0 mmol/L in some samples.

In the other geoparks, the patterns of pollution differ. The Geomining Park of Sardinia demonstrates a high level of arsenic in soils, surface water, and groundwater at a former mining site [43]. Giurginca et al. [52] reported the presence of several potentially toxic elements in leaf litter and invertebrates from the Mehedinti Plateau Geopark; of these, pollution by copper is specific to the study area. In the latter study, the same research team [53] confirmed pollution of soils and cave sediments in this geopark by copper and some accompanying metals (cadmium, lead, etc.).

These lines of evidence imply that environmental pollution is registered in several geoparks, and the available examples are rather bold. The main pollutants are trace metals and metalloids (for instance, arsenic is mentioned commonly), although nitrate pollution and noise pollution were also registered. The strength of pollution differs, but examples of heavy pollution are found in the literature. Many works focus on pollution of surface waters and groundwater, whereas soils, bottom sediments, and living organisms are addressed less frequently. Regarding the geological environment, pollution of groundwater, cave deposits, and former mining sites in general is reported. However, it appears that the considered literature pays more attention to non-geological landscape components, which means geoparks are addressed by environmental researchers as natural parks rather than specific, geology-related establishments.

### 3.3. Sources of Pollution

The possible sources of environmental pollution in geoparks mentioned in the analyzed literature differ significantly. In the Langkawi UNESCO Global Geopark, pollution results from agricultural activities, cement production, sand mining, solid waste storage (landfill leachate), and recreation, including boating, oil spills, and waste disposal [44,45,46,47]. In the Polish part of the Muskauer Faltenbogen/Łuk Mużakowa geopark, the presence of former mining sites and the related acid mine drainage and dewatered waste dumps are responsible for some actual and potential pollution [48,49]. Agricultural activities are considered as a factor of pollution risk in the Naturtejo geopark [50]. Antunes et al. [51] revealed the leading role of the abandoned mine in the pollution of the Terras de Cavaleiros geopark. In the Wudalianchi geopark, Zhang et al. [41] identified two factors of environmental pollution, namely chemical fertilizers (and possibly domestic sewage) and nitrification due to soil erosion linked to stone dam construction. Abandoned mining sites with tailings and landfills facilitate environmental pollution in the Geomining Park of Sardinia [43]. Giurginca et al. [52] and Munteanu et al. [53] proposed that mining and other, unspecified human activities triggered pollution in the Mehedinti Plateau Geopark.

These lines of evidence prove the initial idea of multiple sources of environmental pollution in geoparks (Figure 1). In all cases, pollution unrelated to the geopark takes place. This is either inherited from former human activities (chiefly mining), or it is related to present activities (agricultural, waste storage) directly in the geopark or its vicinity. However, two other situations should be noted. One of these is registered in the Langkawi UNESCO Global Geopark, where recreational, i.e., geopark-related activities, trigger environmental pollution. The latter is both direct (pollution from tourism itself) and indirect (waste storage). Another situation is registered in several geoparks, such as the Geomining Park of Sardinia, where pollution results from abandoned mining sites, which themselves constitute geoheritage. If geoparks represent mining activities, they cannot overlook the theme of their environmental impact. Therefore, the pollution is directly related to unique, heritage values, and it can be labeled provisionally as “unique pollution”. Furthermore, it is notable that, of all the geoparks studied in regard to environmental pollution, only the Langkawi geopark functions with a significant influence on the landscape. Indeed, this does not mean that the other geoparks are “environmentally friendly”—most probably, their recreational pressure on nature has not yet been investigated.

### 3.4. Mitigation Approaches

The majority of the considered literature sources focus on environmental pollution itself, and some of them also bear important information about its mitigation and related prescriptions. Aris et al. [44] suggested optimizing networks of monitoring surface water quality in the Langkawi geopark. For the same geopark, Ibrahim et al. [45] recommended to limit boat trips (limitations on their number per day and/or boat speed), to replant mangrove trees, to limit tourist arrivals to the well-fixed carrying capacity, and to increase the environmental literacy of boat operators and other involved stakeholders. Mokhtar et al. [46] found it important to improve solid waste management in the Langkawi geopark. In addition to monitoring, control, and staff education, several technical solutions, such as installing suitable filtration membranes, were prescribed by these experts. Albuquerque et al. [50] proposed a new methodology for groundwater vulnerability assessment, which is important for finding proper foci for subsequent mitigation of potential pollution in the Naturtejo geopark.

In the Polish part of the Muskauer Faltenbogen/Łuk Mużakowa geopark, the former pits were filled with water to create lakes; this newly formed lacustrine landscape has become well resistant to pollution due to the natural properties of the lake bedrocks [48]. If this partly artificial landscape modification can be understood as a kind of rehabilitation, the latter can be judged successful. In contrast, Antunes et al. [51] documented that remediation of the former mining site has not prevented pollution in the Terras de Cavaleiros geopark, which indicates the failure of the implemented rehabilitation approach.

Cho et al. [27] explained that former pollution and subsequent restoration of the Sihwa Lake in the Hwaseong geopark is a premise for the development of educational programs and improvement in the environmental literacy of geopark visitors, including schoolchildren and students. Mikhailenko and Ruban [29] considered the Don River delta as a unique locality with a self-cleaning environment, which does not allow accumulation of mercury in soils despite ongoing pollution. This uniqueness adds heritage value to this locality and makes the possible geopark ideal for geo-ecological education; the latter may become the principal function of this geopark.

The reviewed knowledge indicates the availability of three principal mitigation approaches in geoparks in regard to environmental pollution. The first approach is direct mitigation to be realized in geoparks (and, most probably, by geoparks’ administrations) via environmental monitoring, tourism limitations, and technical solutions. Importantly, this approach can aim at pollution from geoparks themselves and inherited by them. The second approach is indirect mitigation via eco-education and eco-awareness of both visitors and geopark-related stakeholders. These two pro-environmental opportunities are among the positive consequences of geopark functioning (Figure 1). Additionally, one should note approaches used before geopark creation (past mitigation). Some of these are successful, and some are not. Regardless of their success, their implementation did not depend on geoparks, although administration of the latter may extend and improve these approaches.

## 4. Discussion

The lines of the literary evidence reviewed above imply that environmental pollution in geoparks has been investigated in a rather limited number of cases (Table 1). Nonetheless, this issue seems to be urgent because of three main reasons. First, this pollution can be judged to be significant (and, locally, to be very significant) in half of the geoparks in which it was studied. These include the Langkawi, Muskauer Faltenbogen/Łuk Mużakowa, Terras de Cavaleiros, and Wudalianchi UNESCO Global Geoparks (in Malaysia, Poland, Portugal, and China, respectively), in addition to the Geomining Park of Sardinia (Italy) and the Mehedinti Plateau Geopark (Romania). Second, pollutants and pollution sources are rather diverse; in some cases, these are linked to unique geological features or result from geopark functioning. Third, geoparks offer special instruments to mitigate environmental pollution and to use it for cultivation of pro-environmental behavior. In other words, pollution happens in some geoparks and manifests its specific features.

The scale of the problem is yet to be known due to the lack of investigations. Nonetheless, the example of the Langkawi geopark demonstrates that extensive exploitation of geoheritage resources may cause significant problems, which may be the case in some (if not many) other geoparks created globally. It also appears that environmental pollution results from intersecting interests. Geoparks are created for conservation and efficient use of geoheritage and the hosting landscape, but they may be affected by human activities taking place in neighboring areas or even within geoparks. This means geopark creation requires careful planning.

**Figure 4:**
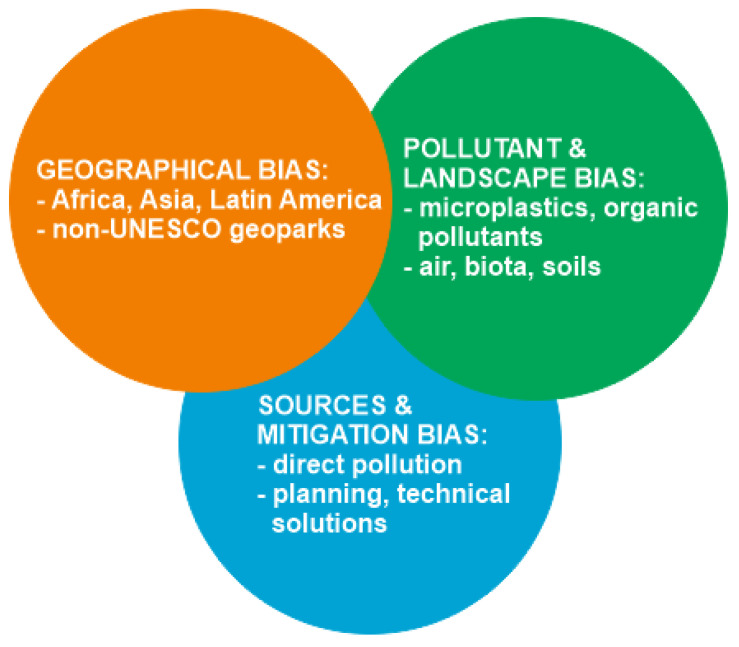
Biases in the current knowledge of environmental pollution in geoparks.

The available knowledge of environmental pollution in geoparks seems to be biased to a significant degree (Figure 4). This knowledge is based on information from several European and a few Asian geoparks (Figure 1), whereas nothing is known about African and Latin American geoparks. Moreover, there are many geoparks in China and other Asian countries [17]; these are exploited intensively for the purposes of tourism, and it is crucial to document pollution (or its absence) from these activities. The examples of the other natural parks and protected areas demonstrate that environmental stress from tourism can be very significant [55,56,57,58,59]. The available knowledge is biased in regard to pollutants and landscape components. Much attention has been paid to trace metals and metalloids, but microplastics are also dangerous and are associated commonly with tourist activities [20,60,61]. Additionally, the natural radioactivity of rocks and groundwater can be related to pollution due to the greater exposure of visitors to geoparks, providing a deeper interaction with the geological environment. Moreover, some geoparks host mining sites where the danger can be even higher. The potential influence of natural rock and groundwater radioactivity on human health is well known [62,63]. Moreover, the information about pollution of air, biota, and soils remains insufficient. Finally, the reviewed literature pays significant attention to pollution unrelated to geoparks, whereas the degree of direct pollution from geopark functioning remains poorly known. The only representative example is the Langkawi geopark, although it seems to be improbable that geoheritage exploitation does not stress landscapes in the other cases. Sustainable planning and the technical side of pollution mitigation are embraced poorly by the considered literature, although the need for them is undisputable in the light of the reported evidence. Apparently, all these biases are interrelated (Figure 4), which indicates the complexity of the unresolved questions.

Taking into account the noted biases, it can be questioned whether some other sources of information in addition to scientific journal articles would help our understanding. The two most evident candidates are governmental and non-governmental reports and media news. Although any detailed examination of such sources is outside the scope of the present work, a tentative search of the Internet shows an apparent absence (most probably, scarcity) of English-language reports concerning the analyzed issue. Few media news reports are available on-line. In one case, the issue of significant, tourism-related environmental stress, and pollution (particularly, water pollution) in the Langkawi archipelago hosting the similarly named UNESCO Global Geopark is noted (https://news.mongabay.com/2018/04/we-are-going-to-self-destruct-development-plans-threaten-malaysian-island-ecosystem/ accessed on 7 April 2022). This matches very well with what is documented in the scientific literature, where this geopark figures as a bold example of environmental pollution (see above). The other case refers to the situation in Sabah (Indonesia), where the Kinabalu Park is planned to become a global geopark (https://www.dailyexpress.com.my/read/4714/sabah-has-the-right-tourism-stuff-/ accessed on 7 April 2022). It is explained that environmental safety is a big concern (this means that anthropogenic stress is expected), and special approaches such as the “Environmental Police” game are introduced for mitigation. The implementation of such initiatives contributes to the knowledge of the possible mitigation approaches.

The current state of the knowledge of environmental pollution in geoparks allows three scenarios of what can happen to be hypothesized (Table 2). Geotourism growth is a desired pattern, but it can be responsible for significant anthropogenic stress. The latter needs to be minimized by efficient environmental management. Apparently, this stress can be recompensed by ensuring that the local anthropogenic activities not related to geoparks are more sustainable, but this option should not be considered as an alternative to environmental management approaches. In fact, due to the complexity of environmental pollution in geoparks (Figure 1) and the peculiarities of the situation in each given geopark, these scenarios (Table 2) are only conceptually meaningful. Nonetheless, the literary evidence treated in the present work implies that the probability of the pessimistic scenario is rather high—at least, in particular geoparks. Due to the very novelty of these establishments and their highly specific essence, their management is highly challenging, and solving this problem can be related to the deep involvement of various policy makers and governmental authorities in geopark development.

Although a large amount of information has yet to be accumulated, the available facts allow specifying several practical recommendations to contemporary geopark managers. First, geoparks should be planned in regard to inherited (for instance, from past mining activities) and geopark-unrelated (for instance, agricultural and industrial activities within or near geoparks) environmental pollution. Attention should be paid to rehabilitation procedures before and after creation, optimal delimitation of the geopark’s area, finding mechanisms of successful interaction of various stakeholders concerning environmental impacts, and establishing monitoring networks. Second, geoparks should be managed so as to avoid direct and indirect pollution. Specifically, this means avoiding over-tourism via establishing limitations and solving the problems linked to waste storage and transportation. Third, the geo-educational potential of geoparks has to be exploited for cultivating pro-environmental behavior. In addition to conservation of geoheritage, providing recreational services, and sustaining local communities with new job and entrepreneurial opportunities, geopark administrations should use the available interpretative facilities for the explanation of the environmental impact of human activities and nature’s vulnerability. Fourth, the present review demonstrates many dimensions and the complexity of the issue of environmental pollution in geoparks, which means the demand for highly professional knowledge. At least, all main stakeholders should have adequate ecological knowledge to sustainably develop geoparks. Such knowledge would also prevent possible disputes between stakeholders with different interests. Fifth, the present state of the information about environmental pollution in geoparks seems to be emerging, and the related research is rather “marginal” in comparison to the “mainstream” investigations of geoparks and geotourism. Therefore, it is urgent to develop and fund scientific programs aimed at the detailed examination of the environmental state of the existing geoparks. Governments and research organizations must place this issue on their agendas.

## 5. Conclusions

The current review of the lines of literary evidence of environmental pollution in geoparks indicates the emerging state, and the general urgency, of this issue, and also allows three general conclusions to be made. First, environmental pollution is registered in several UNESCO Global Geoparks and other geoparks, and can reach high levels. Second, the previous studies registered pollution of surface waters and groundwaters, in addition to some other landscape components; the commonly reported pollutants are trace metals and metalloids, although other pollutants (e.g., nitrates) and forms of pollution (e.g., noise) were also reported. Third, environmental pollution in geoparks needs mitigation, but it also highlights opportunities for eco-education and eco-awareness. Indeed, the available knowledge is strongly biased (Figure 4), and filling the related gaps signifies perspectives for further investigations. The general situation with environmental pollution in the existing geoparks is uncertain. Although environmental pollution is only registered in a small proportion of geoparks, there are examples of significant pollution, which are alarming. Apparently, the true degree of the problem is yet to be fully realized because geoparks are rather new establishments and the researchers have not paid adequate attention to them. However, it is expected that this problem will grow in the near future due to the growth in geopark networks, the increase in global geotourist activities, and the intensification of research aimed at environmental pollution in geoparks.

The present review reveals a new research direction. It can be anticipated that the demand for this research will only rise in the future. This is because more and more geoparks are being created globally; in addition, the previously created geoparks are becoming “mature”, which means their attractiveness among tourists and the diversity of activities with significant environmental impacts are increasing. The number of related journal articles should increase, from the small amount found in this study, to dozens of items published annually. The most promising topics for further investigations include analysis of groups of geoparks with the same set of environmental criteria, finding specific mechanisms of environmental pollution linked to tourist activities in geoparks, and assessing the implemented and planned management strategies in regard to their efficacy in pollution mitigation. In other words, methodological developments and empirical research are demanded. Two main restrictions for the noted research growth are linked to the specific essence of geoparks and the experts’ attention to unique geological features rather than whole landscapes.

Two limitations of the present work are as follows. First, it is based on only literary evidence. Indeed, a systematic review of the latter is essential for the understanding of the state of the problem as revealed by scholars, i.e., it is suitable as the first step towards a comprehensive treatment of the problem. However, other lines of evidence, which can be found in various reports of governmental authorities and non-governmental organizations, or can be deduced from interviews with geopark managers, have to be taken into account. Indeed, this requires new studies, which would be challenging because a part of the related information is available in languages other than English, and the authors of this information are not necessarily competent in either environmental issues or specifics of geoheritage management. Second, it cannot be excluded that some relevant research outcomes were published in national or local journals (or books and abstract volumes), which are not covered by “Scopus” (see description of this issue by Tennant [64]). Unfortunately, this missing information cannot be excluded. However, it is also evident that the outcomes of the most important, world-class research are published in international journals, which are covered by this bibliographical database very well. This information seems to be enough in the present systematic review due to its pioneering nature. Future research can consider some nationally/locally available investigations.

## Figures and Tables

**Table 1:** Summary of the literary evidence considered in the present review (this information is based on the considered literature).

Geopark	Pollution State	Principal Pollutant	Source of Pollution	Mitigation Approach
Apuan Alps (Italy)	absent	-	-	-
Langkawi (Malaysia)	very significant *	heavy metals and metalloids, oils, litter	complex anthropogenic stress, tourism	proposed by scientists
Muskauer Faltenbogen/Łuk Mużakowa (Polish part)	present **	heavy metals and metalloids	inherited	implemented successfully *
Naturtejo (Portugal)	potential	-	agriculture	proposed by scientists
Terras de Cavaleiros (Portugal)	very significant **	heavy metals and metalloids	inherited	implemented and failed *
Wudalianchi (China)	present *	nitrates	complex anthropogenic stress	-
Geomining Park of Sardinia (Italy)	present **^,^***	heavy metals and metalloids	inherited	-
Hwaseong (South Korea)	past ***	-	-	proposed by scientists
Mehedinti Plateau (Romania)	present **	heavy metals and metalloids	complex anthropogenic stress	-
Don River delta (Russia)	self-cleaning environment **	heavy metals and metalloids	complex anthropogenic stress and natural peculiarities	proposed by scientists

Time context: * a few last decades, ** the 20th century, *** historical past (not specified).

**Table 2:** General environmental scenarios for geopark-based geotourism.

Scenario	Geotourism and Related Infrastructure	Local Anthropogenic Activities	Monitoring, Mitigation, Rehabilitation	Pollution
Optimistic	Rising	Sustainable	Permanent and high-quality	Minimal
Neutral	Rising	Sustainable to certain degree	Occasional or chaotic	Stable or slightly rising
Pessimistic	Rising, then stable or dropping	Accelerating	Absent or minimal	Rising

## Data Availability

Not applicable.
